# Spirometry begins before expiration: why acceptability must be reported

**DOI:** 10.36416/1806-3756/e20260147

**Published:** 2026-09-14

**Authors:** Maurício Domingues Ferreira

**Affiliations:** 1. Laboratório de Investigação Médica - LIM-56 - Instituto de Medicina Tropical, Faculdade de Medicina, Universidade de São Paulo, São Paulo (SP) Brasil.

## DEAR EDITOR:

Acceptability in spirometry constitutes the primary determinant of diagnostic validity, preceding any pathophysiological interpretation. Technical flaws may distort results and lead to erroneous physiological interpretations; therefore, verification of maneuver quality is an essential step prior to clinical analysis. Although widely described in guidelines, acceptability criteria remain underestimated in functional reports, even in prevalent conditions such as asthma and COPD or in the differentiation between obstructive and restrictive patterns. In this context, it is essential that acceptability and reproducibility criteria be explicitly reported in spirometry reports as part of the minimum standard of technical quality, particularly when clinical decisions depend on small variations in FEV_1_ and FVC.

The 2024 update from the *Sociedade Brasileira de Pneumologia e Tisiologia* (SBPT, Brazilian Thoracic Association) recommendations places acceptability back at the center of evaluation by emphasizing that the forced maneuver begins with maximal inspiration and proper control of the initial effort, ending only when objective criteria ensure the reliability of FVC and FEV_1_.^1^ Thus, the focus shifts from merely obtaining values to explicitly demonstrating technical validity-a prerequisite for clinical interpretation. Although aligned with international standardization,^2^ ^,^ ^3^ the SBPT guidelines preserve inspection of the flow-volume curve and control of PEF as central elements in quality assessment.^1^

The FVC maneuver comprises four phases: rapid inspiration to TLC, explosive onset of expiration, continuous expiration to plateau (or maximum) time, and a new inspiration back to TLC. Since FVC represents the volume mobilized between TLC and RV, any failure to reach TLC before expiration may underestimate FVC, simulate restriction or a nonspecific pattern, and lead to incorrect functional classifications. In patients with interstitial lung disease under follow-up, submaximal inspiration may falsely suggest longitudinal FVC decline and lead to inappropriate therapeutic escalation when the observed reduction results from technical error. Therefore, it is recommended that forced inspiratory vital capacity (FIVC) and FVC (difference < 100 mL or 5%) be compared and that the alignment of inspiratory and expiratory loops be verified as indirect confirmation of an adequate start of the maneuver.^1^

In cases of significant airflow obstruction, FIVC may exceed FVC due to flow limitation and early airway closure during forced expiration, leading to air trapping and consequent underestimation of FVC, without necessarily implying measurable changes in RV. In patients with COPD, this discrepancy may influence functional classification and prognostic staging, reinforcing the need for contextualized interpretation.

Beyond achieving an adequate start, acceptability requires the absence of hesitation (Chart 1). Two markers summarize this phase. The inspiratory pause before expiration should be ≤ 2 s, as delays may shift the “time zero” and distort FEV_1_ and FVC. Back-extrapolated volume (VExt), corresponding to the volume exhaled before the mathematical definition of time zero, should be ≤ 100 mL or 5% of FVC (whichever is greater), with up to 150 mL considered an acceptable practical limit.^1^ Elevated VExt indicates an inadequate start of expiration and displacement of time zero. This may lead to overestimation of FEV_1_ and alteration of the FEV_1_/FVC ratio, particularly in borderline cases. 

**Chart 1:** Minimum technical criteria required for spirometric acceptability and reproducibility.

Domain	Parameter	Criterion	Clinical consequence if not met
Start of test	Back-extrapolated volume (VBE)	≤ 100 mL or ≤ 5% of FVC (whichever is greater)	Invalid FEV_1_ due to inaccurate time zero definition
Start of test	Explosive start / PEF consistency	Rapid rise to peak flow; ≤ 10% variability between maneuvers	Suggests submaximal effort; compromises flow-dependent indices
End of test	Expiratory duration	Expiratory plateau; if absent, expiratory time ≥ 10 s or FVC within repeatability criteria may be accepted	Underestimation of FVC; risk of false restriction
End of test	Plateau	Change < 25 mL in the final second of expiration	Incomplete emptying; unreliable volume measurement
Artifacts	Cough in the first second	Absent	Invalidates FEV_1_
Artifacts	Glottic closure / early termination	Absent	Underestimation of FVC and distorted curve morphology
Reproducibility	FEV_1_ repeatability	≤ 150 mL between the two highest values	Indicates acceptable measurement consistency
Reproducibility	FVC repeatability	≤ 150 mL between the two highest values	Indicates acceptable volume consistency

VBE: back-extrapolated volume.

Recent evidence shows that the difference between 100 and 150 mL has minimal impact on the reproducibility of FVC and FEV_1_,^4^ supporting a criterion that is both technically rigorous and pragmatically applicable.

The third element of an adequate start is PEF. The SBPT defines time to reach PEF as ≤ 150 ms and recommends variability ≤ 10% of the highest value obtained.^1^ The ERS/ATS 2019 guidelines do not include PEF repeatability as a formal quality criterion,^2^ based on studies predominantly conducted in elderly populations, in which peak flow variability shows a weak correlation with FEV_1_.^5^ The maintenance of this criterion by the SBPT does not represent opposition to international recommendations but rather a delimitation of their clinical applicability in scenarios where initial effort directly influences functional interpretation. In younger populations or in mild-to-moderate obstruction, initial effort may significantly impact FEV_1_; in mild asthma, a submaximal start may lead to underestimation of FEV_1_ and alteration of the FEV_1_/FVC ratio, suggesting nonexistent obstruction or overestimating obstruction severity.

Beyond the start, acceptability depends on the absence of artifacts such as zero-flow errors, leaks, mouthpiece obstruction, or early glottic closure (Chart 1).^1^ Cough within the first second invalidates FEV_1_ but not necessarily FVC; glottic closure after the first second compromises FVC while potentially preserving a usable FEV_1_. The concept of a “usable parameter” reinforces that acceptability is a property specific to each variable, rather than a binary judgment of the maneuver as a whole.

At the end of the maneuver, the SBPT defines the expiratory plateau as a variation ≤ 25 mL in the last second.^1^ In the absence of a plateau, an expiratory time ≥ 10 s is accepted, considering clinical limitations and fatigue, with 15 s as a rarely relevant upper limit. Historically, a minimum fixed time of 6 s in adults (and 3 s in children) was used; however, the 2024 update prioritizes the presence of a plateau as the primary criterion, replacing a rigid temporal threshold with a model centered on effective lung emptying. The absence of a true plateau may imply premature termination of expiration and consequent underestimation of FVC, with potential impacts on functional classification in both obstructive and restrictive disorders. This change reflects a conceptual advance by prioritizing effective lung emptying over arbitrary temporal criteria.

A frequently neglected aspect is the explicit documentation of acceptability and reproducibility criteria in the report (Chart 2). In many laboratories, results are presented without clear indication of the achieved technical quality, limiting critical evaluation by the requesting clinician. The absence of documented technical quality may lead to clinical decisions based on unreliable measurements, with implications for patient safety, avoidable diagnostic errors, and healthcare management and costs. The validity of the test must be demonstrable, auditable, and explicitly communicated in the report.^1^ It is recommended that reports include the following: number of acceptable maneuvers, quality grade (A-F), and measures of reproducibility (ΔFEV_1_ and ΔFVC) as an objective expression of the technical reliability of the results, in addition to the recording of artifacts (Chart 2).

**Chart 2:** Essential elements that must be explicitly reported in spirometry reports.

Domain	Parameter	Description	Clinical implication
Technical quality	Number of acceptable maneuvers	Total maneuvers meeting acceptability criteria	Reflects robustness of test execution
Technical quality	Quality grade (A-F)	Classification based on reproducibility criteria	Defines overall reliability of the test
Reproducibility	Δ FEV_1_	Absolute difference between the two highest FEV1 values	Quantifies variability of airflow measurement
Reproducibility	ΔFVC	Absolute difference between the two highest FVC values	Quantifies variability of volume measurement
Acceptability	Start-of-test quality	VBE, inspiratory pause, and PEF behavior	Ensures validity of initial effort and FEV_1_
Acceptability	End-of-test criteria	Expiratory plateau; if absent, expiratory time ≥ 10 s or FVC within repeatability criteria may be accepted	Confirms complete expiration and valid FVC
Morphology	Flow-volume curve inspection	Visual assessment of loop shape and reproducibility	Detects technical artifacts and physiological inconsistencies
Consistency	FIVC vs. FVC comparison	Difference < 100 mL or < 5%	Confirms adequate inspiration to total lung capacity
Artifacts	Recording of artifacts	Documentation of cough, leaks, or glottic closure	Allows critical appraisal of test validity
Interpretation support	Statement of technical validity	Explicit declaration of whether criteria were met	Prevents interpretation of technically invalid data

Δ: difference between the two best maneuvers; FIVC: forced inspiratory vital capacity; and VBE: back-extrapolated volume.

Compared with the international framework, the SBPT 2024 recommendations preserve classical standardization but explicitly incorporate a philosophy of technical rigor aligned with clinical applicability. By emphasizing inspection of the flow-volume curve and control of PEF, they recognize that small technical flaws may alter FEV_1_ and FVC and modify functional classification. We recommend that spirometry reports systematically include technical quality indicators as a minimum requirement for reliable interpretation. Without such documentation, functional interpretation is no longer fully verifiable. Ultimately, acceptability is not an operational formality-it is a structural condition for diagnostic validity in pulmonary practice.

## References

[1] Albuquerque ALP, Berton DC, Ferreira EVM, Queiroga-Júnior FJP, Santana ANC, Wong BMS (2025). New spirometry recommendations from the Brazilian Thoracic Association - 2024 update. J Bras Pneumol.

[2] Graham BL, Steenbruggen I, Miller MR, Barjaktarevic IZ, Cooper BG, Hall GL (2019). Standardization of Spirometry 2019 Update An Official American Thoracic Society and European Respiratory Society Technical Statement. Am J Respir Crit Care Med.

[3] Miller MR, Hankinson J, Brusasco V (2005). Standardisation of spirometry. Eur Respir J.

[4] Sharifi V, Brazzale DJ, Lanteri CJ, Ruehland WR (2023). The effect of 2019 ATS/ERS criteria for back-extrapolated volume on the acceptability and interpretation of spirometry results is minor. Ann Am Thorac Soc.

[5] Hegewald MJ, Lefor MJ, Jensen RL, Crapo RO, Kritchevsky SB, Haggerty CL (2007). Peak expiratory flow is not a quality indicator for spirometry peak expiratory flow variability and FEV1 are poorly correlated in an elderly population. Chest.

